# Sublingual immunotherapy increases Treg/Th17 ratio in allergic rhinitis

**DOI:** 10.1515/med-2021-0285

**Published:** 2021-05-21

**Authors:** Jiarong Wang, Liansheng Qiu, Yimin Chen, Minyun Chen

**Affiliations:** Department of Otorhinolaryngology, The Second Affiliated Hospital of Fujian Medical University, No. 34 Zhongshan North Road, Quanzhou, Fujiain, 362000, China

**Keywords:** rhinitis, allergic, immunologic, T-lymphocyte regulatory, Th17 cells

## Abstract

**Background:**

Few studies investigated the effects of sublingual immunotherapy (SLIT) on the peripheral regulatory T cells (Tregs)/Th17 ratio.

**Objective:**

To investigate the effectiveness of SLIT in children with allergic rhinitis (AR) and the effects on the Tregs/Th17 ratio.

**Methods:**

This was a retrospective study of children who were treated for AR between April 2017 and March 2018 at one hospital. The patients were grouped according to the treatments they received: SLIT + pharmacotherapy vs pharmacotherapy alone.

**Results:**

Eighty children (51 boys and 29 girls; 40/group) were included. The visual analog scale (VAS) and medication scores at 1 year in the SLIT + pharmacotherapy group were 2.70 ± 1.08 and 1.1 ± 0.8, respectively, which were lower than at baseline (7.7 ± 1.2 and 3.6 ± 1.0, respectively) (both *P*s < 0.05). For the pharmacotherapy group, the VAS score was decreased at 1 year vs baseline (3.3 ± 1.2 vs 7.4 ± 1.0; *P* < 0.05), but the medication score did not change (*P* > 0.05). In the SLIT + pharmacotherapy group, the Treg percentage increased, while the Th17 percentage decreased at 1 year (both *P*s < 0.01). The percentages of Tregs and Th17s did not change in the pharmacotherapy group (both *P*s > 0.05).

**Conclusions:**

SLIT + pharmacotherapy can increase the Treg percentage and decrease the Th17 percentage in the peripheral blood of children with AR.

## 1 Introduction

Allergic rhinitis (AR) is the most common noninfectious inflammatory disease of the nasal mucosa and is mainly mediated by IgE after exposure to allergens [1,2]. The prevalence of AR is very high and still increasing [3,4]. According to a recent study, the average prevalence of AR is as high as 17.6% in 17 central cities in China [5,6]. The increase in AR prevalence in children is even faster than in adults, and AR in some children can progress to bronchial asthma [7].

Allergen-specific immunotherapy (AIT), also known as specific immunotherapy (SIT), is currently the only etiological treatment able to alter the natural course of AR through immunoregulation. AIT improves the clinical and immune tolerance of the patients and reduces the risks of new sensitization and progression from AR to asthma. The effectiveness, long-term effects, and safety profiles of SIT have already been confirmed by various studies [8,9,10,11]. Therefore, this treatment has been recommended by several guidelines, including the Chinese AR diagnosis and treatment guidelines [12], the latest Chinese AIT guidelines [13], and the European AIT guidelines [14]. The most common methods for SIT include subcutaneous immunotherapy (SCIT) and sublingual immunotherapy (SLIT). Although SLIT has already been used in clinical practices for over three decades, it is still a “new” method compared with SCIT, which has over 100 years’ history of clinical application [15,16,17]. Studies have shown that SLIT has evident efficacies for patients with IgE-mediated airway allergic diseases. Due to the good safety profiles and tolerability, SLIT is especially suitable for the treatment of AR and asthma in children [15,16,17].

Previous studies suggested that AR is an allergic inflammation associated with nasal mucosal Th2 immunity caused by the imbalance of Th1/Th2 cells [18]. Regulatory T cells (Tregs) participate in allergic diseases and can suppress the immune responses regulating pathological and physiological events, therefore, leading to autoimmune tolerance and maintenance of immune balance [19]. CD4^+^ CD25^+^ Treg is among the major types of Tregs. CD4^+^ CD25^+^ Tregs secrete cytokines such as TGF-β-1 and IL-10 and receive regulatory signals mainly from Foxp3 [20]. Th17 cells are another group of T cells that play important roles in promoting inflammatory responses and autoimmune diseases. Th17 cells mainly secrete IL-17, and RORγt is the major nuclear transcription factor of Th17 [21,22].

To date, most studies on the efficacy of immunotherapy in children with AR focused on the assessment of the changes in clinical symptoms before and after treatment, while only very few studies investigated the changes in Tregs and Th17 cells. Previous studies have shown that SCIT could induce the production of Treg cells and suppress the proliferation of Th17 cells and that immunotherapy can lead to increase in the Treg/Th17 ratio in the peripheral blood [10,23,24,25]. On the other hand, few studies investigated the effects of SLIT on the peripheral Treg/Th17 ratio. Therefore, the aim of the present study was to investigate the effectiveness of SLIT + pharmacotherapy in children with AR, the effects on the Treg/Th17 ratio, and the underlying mechanisms.

## 2 Methods

### 2.1 Study design and patients

This was a retrospective cohort study of children who were treated between April 2017 and March 2018 at one hospital for symptoms including nasal congestion, sneezing, water-like snot, and rhinocnesmus and diagnosed with perennial AR. This study was approved by the ethics committee of the hospital. The requirement for informed consent was waived by the committee.

The inclusion criteria were (1) 4–14 years of age and (2) diagnosed with AR, with the symptoms of nasal congestion, sneezing, water-like snot, or rhinocnesmus, and combined with skin prick test (SPT) or IgE positivity. The exclusion criteria were (1) severe or uncontrolled bronchial asthma, (2) treatment with β-blocker, (3) severe cardiovascular diseases, (4) any other immunological diseases or immunodeficiency diseases, (5) systemic or local glucocorticoid therapy within the last month, or (6) unavailable clinical data or lost to follow-up.

AR was diagnosed according to the Tianjin Guidelines for the Diagnosis and Treatment of Allergic Rhinitis (2015, Tianjin) [26]. The children were divided into the SLIT + pharmacotherapy group (received SLIT with standardized dust mite drops combined with pharmacotherapy) and pharmacotherapy group, according to the treatments they received. The main reasons why some parents did not choose SLIT include a lack of understanding of the advantages, risks, and limitations of SLIT, concerns with the financial burden, and lack of compliance over 1–2 years. The children were matched 1:1 according to age and gender.

### 2.2 Treatment methods

For the children in the pharmacotherapy group, the second-generation antihistamines (oral, once per day) and nasal corticosteroids (nasal spray, 1–2 times per day) were administrated for conventional pharmacotherapy according to the Tianjin guidelines (2015) [12].

For the children in the SLIT + pharmacotherapy group, conventional pharmacotherapy combined with SLIT of standardized dust mite drops (No. 1–4, Changdi®; Wolw Pharmaceuticals, Zhejiang, China) was given for SIT. In the increasing dose period (weeks 1–3), Changdi® No. 1–3 was applied (protein concentrations of 1, 10, and 100 μg/mL, respectively; the doses during a week were 1, 2, 3, 4, 6, 8, and 10 drops, respectively, once per day). Changdi® No. 4 was administrated starting on the 4th week (protein concentration of 333 μg/mL, once per day, 3 drops/administration, sublingual administration). Conventional pharmacotherapy based on antihistamines and nasal corticosteroids was also applied. The children were treated for 1 year.

### 2.3 Clinical assessment

The visual analog scale (VAS) score was used to determine the severity of the symptoms. The VAS score ranges from 0 to 10. An analog scale of 10 cm was used for scoring, with 0 indicating no symptom and 10 indicating the most severe symptoms. The children or the guardians marked the corresponding score on the scale according to the symptoms in the last week.

The use of antihistamines and nasal corticosteroids were scored by the children. The score ranged from 0 to 5: where 0 indicated no need for medication; 1 indicated occasional use of medication; 2 indicated often using medication; 3 indicated using medication almost every day; 4 indicated using medication every day; and 5 indicated using medication every day and at the highest dose.

### 2.4 Flow cytometry

Peripheral venous blood (2 mL) was obtained from all children before and at 12 months of treatment, using an aseptic technique. Flow cytometry for Th17 cells was conducted using the Th17 kit from eBioscience, Inc. (San Diego, CA, USA) according to the instructions of the manufacturers. The PerFix-NC test kit (no centrifuge assay kit; Beckman Coulter, Brea, CA, USA) was used for the staining of IL-17 according to the instructions of the manufacturers. CD3^+^ CD4^+^ IL-17^+^ cells were considered Th17 cells. CD4^+^ CD25^+^ CD127^low^ cells were considered Treg cells.

### 2.5 Follow-up

All children were routinely followed by telephone or SMS once per month. The follow-ups included the improvement in symptoms, nasal signs, and drug use. The drugs for the following month were also prescribed, and the children were informed to come back to the hospital for blood examinations after 12 months of treatment. All children were followed for at least 1 year. The children lost to follow-up were not included in the analysis.

### 2.6 Statistical analysis

SPSS 21.0 (IBM, Armonk, NY, USA) was used for statistical analysis. Categorical data were presented as *n* (%). Continuous data were tested for normal distribution using the Kolmogorov–Smirnov test. Normally distributed data were presented as means ± standard deviations and were tested using Student’s *t* test (intergroup comparisons) or the paired *t* test (pre-/posttreatment comparisons). Continuous data with a skewed distribution were presented as medians (interquartile ranges) and were analyzed using the rank-sum test (intergroup comparisons) or the signed-rank test (pre-/posttreatment comparisons). Two-sided *P* values < 0.05 were considered statistically significant.

## 3 Results

### 3.1 Characteristics of the patients

Eighty children (51 boys and 29 girls) were included in this study. They were 8.0 ± 2.5 years of age. Age, VAS score, medication score, Th17 percentage, and Treg percentage before treatment were not significantly different between the SLIT + pharmacotherapy and pharmacotherapy groups (all *P*s > 0.05; Table 1).

**Table 1:** Baseline data according to the SLIT + pharmacotherapy and pharmacotherapy groups

Variable	SLIT + pharmacotherapy (*n* = 40)	Pharmacotherapy (*n* = 40)	*P*
Male, *n* (%)	24 (60.0%)	27 (67.5%)	0.487
Age (years)	8.1 ± 2.5	7.8 ± 2.5	0.480
VAS score	7.7 ± 1.2	7.4 ± 1.0	0.266
Medication score	3.6 ± 1.0	3.6 ± 0.6	0.89
Th17 (%)	1.54 ± 0.41	1.71 ± 0.38	0.10
Treg (%)	7.26 ± 0.92	7.32 ± 1.06	0.80

All continuous data were normally distributed and are presented as means ± standard deviation.

### 3.2 VAS score and medication score

Table 2 showed the VAS score of the AR symptoms and the medication score in the two groups before and after treatment. For the children in the SLIT + pharmacotherapy group, the VAS and medication scores after 6 and 12 months of treatment were significantly different from the baseline scores (all *P*s < 0.05). For the children in the pharmacotherapy group, the VAS scores at 6 and 12 months were also significantly different from the baseline score (both *P*s < 0.05), but the medication score did not change significantly (both *P*s > 0.05).

**Table 2:** Comparisons of the VAS score of AR symptoms and medication score before and after treatment in two groups

Variable	SLIT + pharmacotherapy (*n* = 40)	*P* (pre-/posttreatment comparisons)	Pharmacotherapy (*n* = 40)	*P* (pre-/posttreatment comparisons)	*P* (between groups)
**VAS score**					
Before	7.7 ± 1.2		7.4 ± 1.0		0.266
6 months	2.7 ± 1.1	<0.01	3.5 ± 1.2	<0.01	0.827
1 year	2.7 ± 1.1	<0.01	3.3 ± 1.2	<0.01	0.022
**Medication score**					
Before	3.6 ± 1.0		3.6 ± 0.6		0.893
6 months	2.0 ± 0.9	<0.01	3.5 ± 0.7	0.58	<0.01
1 year	1.1 ± 0.8	<0.01	3.5 ± 0.9	0.43	<0.01

All continuous data were normally distributed and are presented as means ± standard deviation.

### 3.3 Treg and Th17 cells

**Figure 1:**
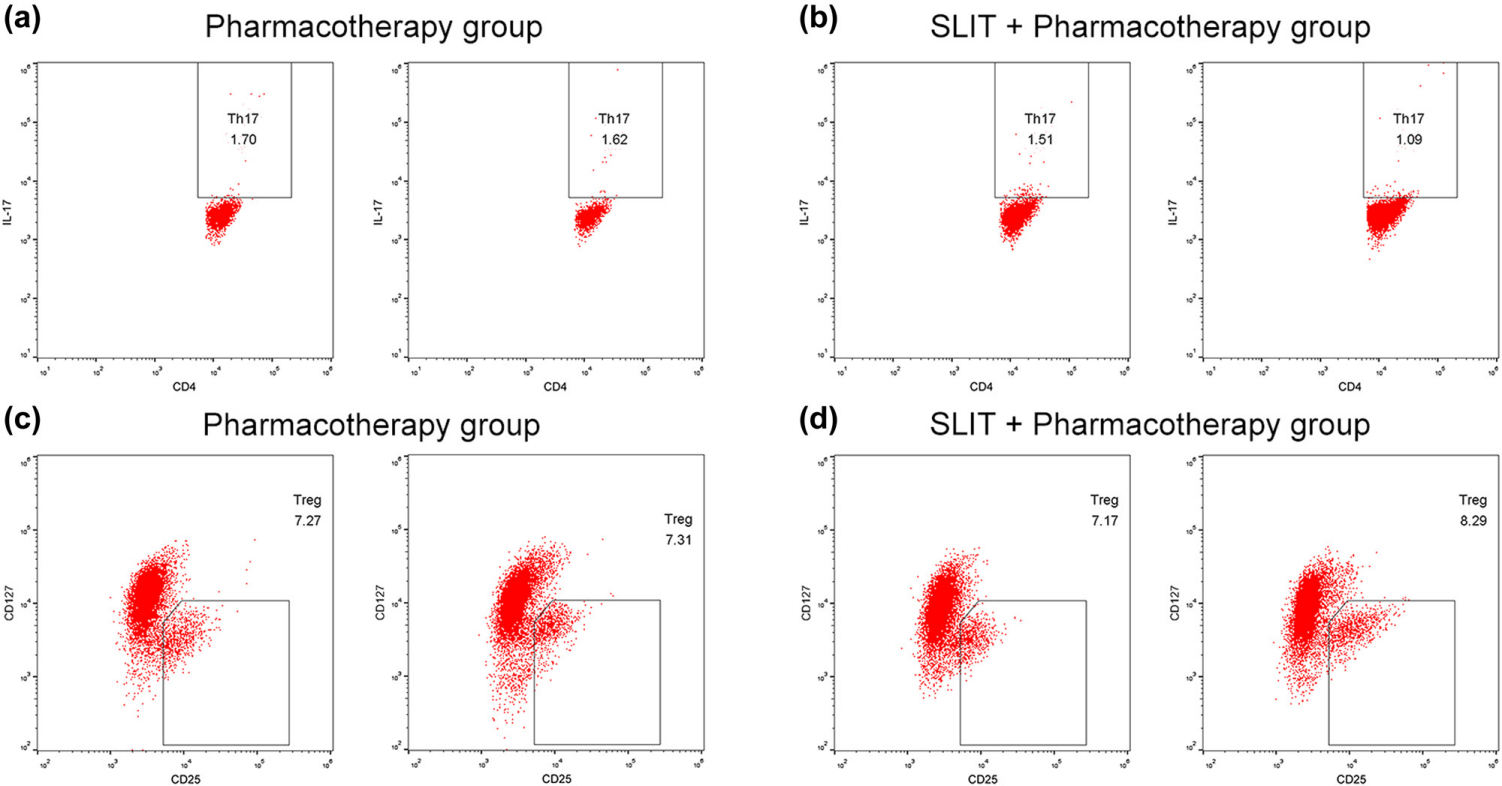
Representative flow cytometry dot plots for Th17 and Treg cells in the SLIT + pharmacotherapy and pharmacotherapy groups after 1 year of treatment. (a) Th17 cells before and after treatment in the pharmacotherapy group. (b) Th17 cells before and after treatment in the SLIT + pharmacotherapy group. (c) Treg cells before and after treatment in the pharmacotherapy group. (d) Treg cells before and after treatment in the SLIT + pharmacotherapy group.

The Treg percentage in the peripheral blood in the SLIT + pharmacotherapy group increased after treatment, compared with baseline (from 7.26 ± 0.92% to 8.23 ± 0.85%; *P* < 0.01) but remained unchanged in the pharmacotherapy group (*P* = 0.68). In contrast, the Th17 percentage in the peripheral blood in the SLIT + pharmacotherapy group decreased after treatment, compared with baseline (from 1.54 ± 0.41% to 1.06 ± 0.21%; *P* < 0.01) but remained unchanged in the pharmacotherapy group (*P* = 0.28). The Treg/Th17 ratio increased after treatment in the SLIT + pharmacotherapy group (from 5.06 ± 1.56% to 8.09 ± 2.00%; *P* < 0.01) but did not change significantly in the pharmacotherapy group (*P* = 0.54; Table 3). The representative dot plot images for Th17 and Treg cells in the SLIT + pharmacotherapy and pharmacotherapy groups are shown in Figure 1.

**Table 3:** Comparisons of the Treg and Th17 percentages in peripheral blood before and after treatment in two groups

Variable	SLIT + pharmacotherapy (*n* = 40)	*P* (pre-/posttreatment comparisons)	Pharmacotherapy (*n* = 40)	*P* (pre-/posttreatment comparisons)	*P* (between groups)
**Treg (%)**					
Before treatment	7.26 ± 0.92		7.32 ± 1.06		0.794
1 year after treatment	8.23 ± 0.85	<0.01	7.41 ± 0.79	0.68	<0.01
**Th17 (%)**					
Before treatment	1.54 ± 0.41		1.71 ± 0.38		0.097
1 year after treatment	1.06 ± 0.21	<0.01	1.64 ± 0.94	0.28	<0.01
**Treg/Th17 ratio**					
Before treatment	5.06 ± 1.56		4.40 ± 1.38		0.061
1 year after treatment	8.09 ± 2.00	<0.01	4.55 ± 0.63	0.54	<0.01

## 4 Discussion

Few studies investigated the effects of SLIT on the peripheral Tregs/Th17 ratio in children with AR. This study aimed to investigate the effectiveness of SLIT + pharmacotherapy in children with AR, the effects on Tregs/Th17 cells, and the underlying mechanisms. The results suggested that SLIT + pharmacotherapy can increase the Treg percentage and decrease the percentage of Th17 in the peripheral blood of children with AR. The symptoms of the children with AR in both SLIT + pharmacotherapy and pharmacotherapy groups were improved by the respective treatments.

The treatments of AR in children mainly include symptomatic pharmacotherapy and SIT. This study showed that after 1 year of SLIT + pharmacotherapy treatment, the VAS and medication scores of the children significantly decreased compared with the baseline. On the other hand, for the children who received only pharmacotherapy, the VAS score decreased compared with the baseline, but the medication score did not significantly change. These findings strongly suggest that the application of either pharmacotherapy alone or pharmacotherapy in combination with SLIT could improve the clinical symptoms of AR children in certain degrees. Although antihistamines and nasal corticosteroids could effectively control the AR symptoms, they could not treat the disease, and thus long-term medication was required for children. In contrast, concurrent treatment with SLIT for 1 year not only reduced the consumption of medication but also resulted in the induction of peripheral immune tolerance and thus could be used as a strategy for the etiological treatment of AR. The underlying cellular and immune mechanisms were then explored.

The immune events causing the development and progression of AR are very complex, involving Th1, Th2, Tregs, and Th17 cells. IL-17 (secreted by Th17) is a pro-inflammatory cytokine that can activate pro-inflammatory factors and regulate IL-5, a Th2 cytokine, regulating the development and progression of AR [10,24,25]. In addition, IL-17 can induce the expression of chemokines (such as monocyte chemoattractant protein-1 and macrophage inflammatory protein-2) and matrix metalloproteinase, which led to immune cell infiltration and inflammation [27]. Qu et al. [28] showed that SIT could downregulate the Th17 percentage in patients with AR, the mRNA levels of the transcription factor RORγt receptor, and the expression of cytokines, including IL-6, IL-17, and IL-23. In the present study, Th17 cells decreased after SLIT but not after pharmacotherapy alone.

Tregs are a group of CD4^+^ CD25^+^ T cells that have negative regulatory effects on inflammation and immune activation. Foxp3 is the specific marker of Tregs and participates in the differentiation and development of Tregs [29]. Tregs secrete cytokines such as IL-10 to induce immune tolerance [30], suppress immune responses of auto-reactive T cells, inhibit T-cell activation, and promote the secretion of several inhibitory cytokines and thus play important roles in the maintenance of peripheral immune tolerance [31]. SLIT aims to induce the establishment of peripheral T-cell tolerance, regulate the threshold of the activation of mast cells and basophils, and reduce the release of IgE-mediated histamines [24]. The induction of peripheral T-cell tolerance is the key mechanism of SLIT. Peripheral T-cell tolerance is characterized by the production of antigen-specific Treg cells, which could produce anti-inflammatory factors such as IL-10 and TGF-β. Tregs not only downregulate the activity of Th2 cells but also directly inhibit the functions of effector cells such as dendritic cells, mast cells, eosinophil, and basophils. Meiler et al. [32] demonstrated that Tregs regulate the level of antigen-specific IgE and induce the production of IgG and IgA [32]. The present study showed that treating patients having dust mite allergy with SLIT + pharmacotherapy for 1 year could increase the Treg percentage, which is in agreement with the changes in the clinical symptoms and the changes in Th17.

Previous studies showed that the production of Tregs and Th17s is induced by TGF-β, but that naive CD4^+^ T cells can be induced to differentiate into Tregs only in the presence of TGF-G. On the other hand, the presence of both TGF-β and IL-6 could coinduce the differentiation of CD4^+^ T cells into Th17 cells. These findings demonstrate the reciprocal inhibition between the differentiation of Th17s and Tregs. In normal conditions, a balance can be observed between the two cell types [33,34,35]. It has been shown that compared with healthy controls, adult patients with AR display higher levels of Th17 cells and reduced Treg cells [36,37]. Similar result has also been found in children with AR [38,39]. This study showed that SLIT could induce the production of Treg cells and inhibit the proliferation of Th17 cells. The Treg/Th17 ratio in the peripheral blood of children with AR increased after SLIT, but this ratio did not change significantly in the children who received only pharmacotherapy. Therefore, it can be speculated that the treatment effectiveness of SLIT + pharmacotherapy on AR could be caused by the upregulation of the Treg/Th17 ratio, which mediated the recovery of the Treg and Th17 cell percentages and functions.

The present study had limitations. The study was retrospective, and the available data were limited to those available in the charts. Only the percentages of Tregs and Th17 cells were examined, while the cytokine levels were not. Finally, SCIT and SLIT were not directly compared. Additional studies are necessary to determine the exact mechanisms of SLIT.

In conclusion, this study examined the Treg and Th17 percentages in the peripheral blood of children with AR after SLIT + pharmacotherapy treatment and assessed the symptom and medication scores. The findings showed that SLIT and pharmacotherapy both improved the symptoms in children with AR. SLIT could reduce the medication administered to children while exerting a better treatment effect than pharmacotherapy alone. SLIT + pharmacotherapy significantly reduced the Th17 percentage in the peripheral blood and increased the Treg percentage. These findings provide data about the cellular immunity in AR before and after immunotherapy, strongly suggesting that SLIT + pharmacotherapy, similar to SCIT, was also effective for the treatment of AR in children.

## References

[1] Seidman MD, Gurgel RK, Lin SY, Schwartz SR, Baroody FM, Bonner JR, et al. Clinical practice guideline: allergic rhinitis. Otolaryngol Head Neck Surg. 2015;152(1 Suppl):S1–43. 10.1177/0194599814561600, 10.1177/0194599814559898. PubMed PMID: 25644617. 25644617

[2] Wallace DV, Dykewicz MS, Bernstein DI, Blessing-Moore J, Cox L, Khan DA, et al. The diagnosis and management of rhinitis: an updated practice parameter. J Allergy Clin Immunol. 2008;122(2 Suppl):S1–84. 10.1016/j.jaci.2008.06.003. PubMed PMID: 18662584. 18662584

[3] Chong SN, Chew FT. Epidemiology of allergic rhinitis and associated risk factors in Asia. World Allergy Organ J. 2018;11(1):17. 10.1186/s40413-018-0198-z. PubMed PMID: 30128063. PubMed Central PMCID: PMC6091170. PMC6091170 30128063

[4] Mims JW. Epidemiology of allergic rhinitis. Int Forum Allergy Rhinol. 2014;4(Suppl 2):S18–20. 10.1002/alr.21385. PubMed PMID: 25182349. 25182349

[5] Zhang Y, Zhang L. Increasing prevalence of allergic rhinitis in China. Allergy Asthma Immunol Res. 2019;11(2):156–69. 10.4168/aair.2019.11.2.156. PubMed PMID: 30661309. PubMed Central PMCID: PMC6340797. PMC6340797 30661309

[6] Zhang Y, Zhang L. Prevalence of allergic rhinitis in china. Allergy Asthma Immunol Res. 2014;6(2):105–13. 10.4168/aair.2014.6.2.105. PubMed PMID: 24587945. PubMed Central PMCID: PMC3936037. PMC3936037 24587945

[7] Gentile D, Bartholow A, Valovirta E, Scadding G, Skoner D. Current and future directions in pediatric allergic rhinitis. J Allergy Clin Immunol. 2013;1(3):214–26 (quiz 27). 10.1016/j.jaip.2013.03.012. PubMed PMID: 24565478. 24565478

[8] Moote W, Kim H. Allergen-specific immunotherapy. Allergy Asthma Clin Immunol. 2011;7(Suppl 1):S5. 10.1186/1710-1492-7-S1-S5. PubMed PMID: 22166078. PubMed Central PMCID: PMC3245438. PMC3245438 22166078

[9] Niederberger V. Allergen-specific immunotherapy. Immunol Lett. 2009;122(2):131–3. 10.1016/j.imlet.2008.11.012. PubMed PMID: 19100771. 19100771

[10] Fujita H, Soyka MB, Akdis M, Akdis CA. Mechanisms of allergen-specific immunotherapy. Clin Transl Allergy. 2012;2(1):2. 10.1186/2045-7022-2-2. PubMed PMID: 22409879. PubMed Central PMCID: PMC3395833. PMC3395833 22409879

[11] Calderon MA, Alves B, Jacobson M, Hurwitz B, Sheikh A, Durham S. Allergen injection immunotherapy for seasonal allergic rhinitis. Cochrane Database Syst Rev. 2007;1:CD001936. 10.1002/14651858.CD001936.pub2. PubMed PMID: 17253469. PMC7017974 17253469

[12] Subspecialty group of rhinology EBoCJoOH, neck S. Subspecialty group of rhinology SoOH, neck surgery CMA. Chinese guidelines for diagnosis and treatment of allergic rhinitis. Chin J Otorhinolaryngol Head Neck Surg (Zhonghua er bi yan hou tou jing wai ke za zhi). 2016;51(1):6–24. 10.3760/cma.j.issn.1673-0860.2016.01.004. PubMed PMID: 26791765. 26791765

[13] Bao Y, Chen J, Cheng L, Guo Y, Hong S, Kong W, et al. Chinese Guideline on allergen immunotherapy for allergic rhinitis. J Thorac Dis. 2017;9(11):4607–50. 10.21037/jtd.2017.10.112. PubMed PMID: 29268533. PubMed Central PMCID: PMC5721020. PMC5721020 29268533

[14] Roberts G, Pfaar O, Akdis CA, Ansotegui IJ, Durham SR, Gerth van Wijk R, et al. EAACI guidelines on allergen immunotherapy: allergic rhinoconjunctivitis. Allergy. 2018;73(4):765–98. 10.1111/all.13317. PubMed PMID: 28940458. 28940458

[15] Canonica GW, Cox L, Pawankar R, Baena-Cagnani CE, Blaiss M, Bonini S, et al. Sublingual immunotherapy: world allergy organization position paper 2013 update. World Allergy Organ J. 2014;7(1):6. 10.1186/1939-4551-7-6. PubMed PMID: 24679069. PubMed Central PMCID: PMC3983904. PMC3983904 24679069

[16] Radulovic S, Calderon MA, Wilson D, Durham S. Sublingual immunotherapy for allergic rhinitis. Cochrane Database Syst Rev. 2010;12:CD002893. 10.1002/14651858.CD002893. PubMed PMID: 21154351. PMC7001038 21154351

[17] James C, Bernstein DI. Allergen immunotherapy: an updated review of safety. Curr Opin Allergy Clin Immunol. 2017;17(1):55–9. 10.1097/ACI.0000000000000335. PubMed PMID: 27906697. PubMed Central PMCID: PMC5644500. PMC5644500 27906697

[18] Agrawal DK, Shao Z. Pathogenesis of allergic airway inflammation. Curr Allergy Asthma Rep. 2010;10(1):39–48. 10.1007/s11882-009-0081-7. PubMed PMID: 20425513. PubMed Central PMCID: PMC2894992. PMC2894992 20425513

[19] Ozdemir C, Akdis M, Akdis CA. T regulatory cells and their counterparts: masters of immune regulation. Clin Exp Allergy. 2009;39(5):626–39. PubMed PMID: 19422105. 10.1111/j.1365-2222.2009.03242.x 19422105

[20] Fehervari Z, Sakaguchi S. Development and function of CD25+ CD4+ regulatory T cells. Curr Opin Immunol. 2004;16(2):203–8. 10.1016/j.coi.2004.01.004. PubMed PMID: 15023414. 15023414

[21] Harrington LE, Hatton RD, Mangan PR, Turner H, Murphy TL, Murphy KM, et al. Interleukin 17-producing CD4+ effector T cells develop via a lineage distinct from the T helper type 1 and 2 lineages. Nat Immunol. 2005;6(11):1123–32. 10.1038/ni1254. PubMed PMID: 16200070. 16200070

[22] Ivanov II, McKenzie BS, Zhou L, Tadokoro CE, Lepelley A, Lafaille JJ, et al. The orphan nuclear receptor RORgammat directs the differentiation program of proinflammatory IL-17+ T helper cells. Cell. 2006;126(6):1121–33. 10.1016/j.cell.2006.07.035. PubMed PMID: 16990136. 16990136

[23] Maggi E. T-cell responses induced by allergen-specific immunotherapy. Clin Exp Immunol. 2010;161(1):10–8. 10.1111/j.1365-2249.2010.04148.x. PubMed PMID: 20408857. PubMed Central PMCID: PMC2940143. PMC2940143 20408857

[24] Akdis CA, Akdis M. Mechanisms of allergen-specific immunotherapy. J Allergy Clin Immunol. 2011;127(1):18–27 (quiz 8–9). 10.1016/j.jaci.2010.11.030. PubMed PMID: 21211639. 21211639

[25] Akdis M, Akdis CA. Mechanisms of allergen-specific immunotherapy: multiple suppressor factors at work in immune tolerance to allergens. J Allergy Clin Immunol. 2014;133(3):621–31. 10.1016/j.jaci.2013.12.1088. PubMed PMID: 24581429. 24581429

[26] Subspecialty group of rhinology EBoCJoOH, neck S. Subspecialty group of R, pediatrics SoOH, neck surgery CMA. Editorial Board of Chinese Journal of P. Guidelines for diagnosis and treatment of pediatric allergic rhinitis (2010, Chongqing). Chin J Otorhinolaryngol Head Neck Surg (Zhonghua er bi yan hou tou jing wai ke za zhi). 2011;46(1):7–8. PubMed PMID: 21429322. 21429322

[27] Wei P, Hu GH, Kang HY, Yao HB, Kou W, Liu H, et al. An aryl hydrocarbon receptor ligand acts on dendritic cells and T cells to suppress the Th17 response in allergic rhinitis patients. Lab Invest. 2014;94(5):528–35. 10.1038/labinvest.2014.8. PubMed PMID: 24514067. 24514067

[28] Qu SH, Li M, Huang YJ, Ou ZY, Lin ZB, Liang JP, et al. Effects of allergen and intranasal glucocorticoid on Th17 and RORgamma t in peripheral blood in patients with allergic rhinitis. Chin J Otorhinolaryngol Head Neck Surg (Zhonghua er bi yan hou tou jing wai ke za zhi). 2009;44(12):996–1000. PubMed PMID: 20193613. 20193613

[29] Ziegler SF, Buckner JH. FOXP3 and the regulation of Treg/Th17 differentiation. Microb Infect. 2009;11(5):594–8. 10.1016/j.micinf.2009.04.002. PubMed PMID: 19371792. PubMed Central PMCID: PMC2728495. PMC2728495 19371792

[30] Peterson RA. Regulatory T-cells: diverse phenotypes integral to immune homeostasis and suppression. Toxicol Pathol. 2012;40(2):186–204. 10.1177/0192623311430693. PubMed PMID: 22222887. 22222887

[31] Corthay A. How do regulatory T cells work? Scand J Immunol. 2009;70(4):326–36. 10.1111/j.1365-3083.2009.02308.x. PubMed PMID: 19751267. PubMed Central PMCID: PMC2784904. PMC2784904 19751267

[32] Meiler F, Klunker S, Zimmermann M, Akdis CA, Akdis M. Distinct regulation of IgE, IgG4 and IgA by T regulatory cells and toll-like receptors. Allergy. 2008;63(11):1455–63. 10.1111/j.1398-9995.2008.01774.x. PubMed PMID: 18925882. 18925882

[33] Zheng SG. Regulatory T cells vs Th17: differentiation of Th17 versus Treg, are the mutually exclusive? Am J Clin Exp Immunol. 2013;2(1):94–106. PubMed PMID: 23885327. PubMed Central PMCID: PMC3714204. PMC3714204 23885327

[34] Gao Z, Gao Y, Li Z, Chen Z, Lu D, Tsun A, et al. Synergy between IL-6 and TGF-beta signaling promotes FOXP3 degradation. Int J Clin Exp Pathol. 2012;5(7):626–33. PubMed PMID: 22977658. PubMed Central PMCID: PMC3438759. PMC3438759 22977658

[35] Kimura A, Kishimoto T. IL-6: regulator of Treg/Th17 balance. Eur J Immunol. 2010;40(7):1830–5. 10.1002/eji.201040391. PubMed PMID: 20583029. 20583029

[36] Huang X, Chen Y, Zhang F, Yang Q, Zhang G. Peripheral Th17/Treg cell-mediated immunity imbalance in allergic rhinitis patients. Braz J Otorhinolaryngol. 2014;80(2):152–5. 10.5935/1808-8694.20140031. Epub 2014/05/17PubMed PMID: 24830974. PMC9443953 24830974

[37] Wilson RH, Whitehead GS, Nakano H, Free ME, Kolls JK, Cook DN. Allergic sensitization through the airway primes Th17-dependent neutrophilia and airway hyperresponsiveness. Am J Respir Crit Care Med. 2009;180(8):720–30 (Epub 2009/08/08). 10.1164/rccm.200904-0573OC. PubMed PMID: 19661246. PubMed Central PMCID: PMCPMC2778149. PMC2778149 19661246

[38] Huang F, Yin JN, Wang HB, Liu SY, Li YN. Association of imbalance of effector T cells and regulatory cells with the severity of asthma and allergic rhinitis in children. Allergy Asthma Proc. 2017;38(6):70–7 (Epub 2017/10/20). 10.2500/aap.2017.38.4076. PubMed PMID: 29046188. 29046188

[39] Kerzel S, Dehne J, Rogosch T, Schaub B, Maier RF, Zemlin M. Th17 cell frequency in peripheral blood from children with allergic asthma correlates with the level of asthma control. J Pediatr. 2012;161(6):1172–4 (Epub 2012/09/05). 10.1016/j.jpeds.2012.07.051. PubMed PMID: 22944005. 22944005

